# A Spacecraft Electrical Characteristics Multi-Label Classification Method Based on Off-Line FCM Clustering and On-Line WPSVM

**DOI:** 10.1371/journal.pone.0140395

**Published:** 2015-11-06

**Authors:** Ke Li, Yi Liu, Quanxin Wang, Yalei Wu, Shimin Song, Yi Sun, Tengchong Liu, Jun Wang, Yang Li, Shaoyi Du

**Affiliations:** 1 Ergonomics and Environment Control Laboratory, Beihang University, Beijing, China; 2 China Academy of Space Technology, Beijing, China; 3 School of Automation Science and Electrical Engineering, Beihang University, Beijing, China; 4 Institute of Artificial Intelligence and Robotics, Xian Jiaotong University, Xian Shanxi Province, China; Nanjing University of Aeronautic and Astronautics, CHINA

## Abstract

This paper proposes a novel multi-label classification method for resolving the spacecraft electrical characteristics problems which involve many unlabeled test data processing, high-dimensional features, long computing time and identification of slow rate. Firstly, both the fuzzy c-means (FCM) offline clustering and the principal component feature extraction algorithms are applied for the feature selection process. Secondly, the approximate weighted proximal support vector machine (WPSVM) online classification algorithms is used to reduce the feature dimension and further improve the rate of recognition for electrical characteristics spacecraft. Finally, the data capture contribution method by using thresholds is proposed to guarantee the validity and consistency of the data selection. The experimental results indicate that the method proposed can obtain better data features of the spacecraft electrical characteristics, improve the accuracy of identification and shorten the computing time effectively.

## I. Introduction

Spacecraft electronic load systems are typically non-linear time-dependent systems, which are complex and uncertain [1–6]. Internal load signal superposition and mutation happen frequently, therefore, the causes of the accident may be intertwined when load system accident occurs [7–12]. If there are no reliable information sources or analysis methods for interpreting the processes of spacecraft electronic load systems, apart from assumptions and speculation, it is challenge to detect the fault of load systems accurately [13–18]. In general, spacecraft electrical characteristics are including voltage signals and current signals, which represent physical processes such as roll, pitch, yaw, voltage fault and current fault [19–22]. During the operation of spacecraft electronic load system, many complex signals could be produced. therefore, it is a challenge problem to detect the fault from the large amount of data from the spacecraft electronic load system. Recently, extensive researches about the electrical characteristics signal identification problem have been conducted [23–27]. For example, Steven et al. analyses nonlinear power systems by using the power system voltage detection [28]. Wang, et al. integrated the characteristics of the spacecraft thermal fault with the idea of artificial intelligence, and the problem was resolved by using a suitable spacecraft fault diagnosis reasoning machine. Thus, the expert system of spacecraft thermal fault diagnosis was established [29, 30]. However, these methods aforementioned need a high precision signal, and thus it is difficult to guarantee the accuracy of complex signal identification. And such methods need a high precision signal, and it is difficult to guarantee the accuracy of complex signal recognition using classic method to resolve multi-label problems [1–6][29, 30]. Additionally, few studies have focused on on-line system classification. Fault identification remains a challenge problem in spacecraft electronic load systems. Other problems including the spacecraft electrical characteristics identification process unlabeled test data, the high dimensional characteristics, the slow computing speed and the low recognition rate should also be solved.

This paper proposes an expert training method to assist the on-line identification system. The method is including two parts: the off-line clustering and the on-line identification part. Firstly, a dataset could be obtained from the off-line system by analyzing the historical data, which the dataset combines objective clustering data and obtains preliminary category labels. Secondly, the search space of these data can be reduced based on a priori information. Furthermore, the preliminary category label samples and filter operation are used to establish the training dataset. Based on the training dataset, an on-line identification algorithm using principal component analysis (PCA) feature extraction and a weighted proximal support vector machine (WPSVM) are proposed to build a better classification learning model. Finally, the experimental data are used to validate the effectiveness of the proposed method.

This paper is organized as follows: Section II identifies the standard of similarity and introduces the fuzzy clustering methods to obtain the training dataset. Next, Section III aims to solve the high dimensional characteristics problems of the training dataset, the principal component analysis is also introduced, and then the data capture contribution algorithm is discussed; And a better classification learning model by using a weighted proximal support vector machine (WPSVM) is proposed. Experimental results are presented in Section IV. Finally, the conclusion is given in Section V.

## II. Off-line system and pretreatment algorithm

The essence of the spacecraft electrical characteristics identification is to contrast the test signal with the standard signal. Thus the error function can be used to evaluate the performance of identification. How to contrast the test signal with the standard signal and how to obtain the standard signal from complex signals are two primary problems in the identification of spacecraft electrical characteristics. First of all, the standard should be defined to contrast with the signals.

### A.Identification of the standard similarity

**Fig 1:**
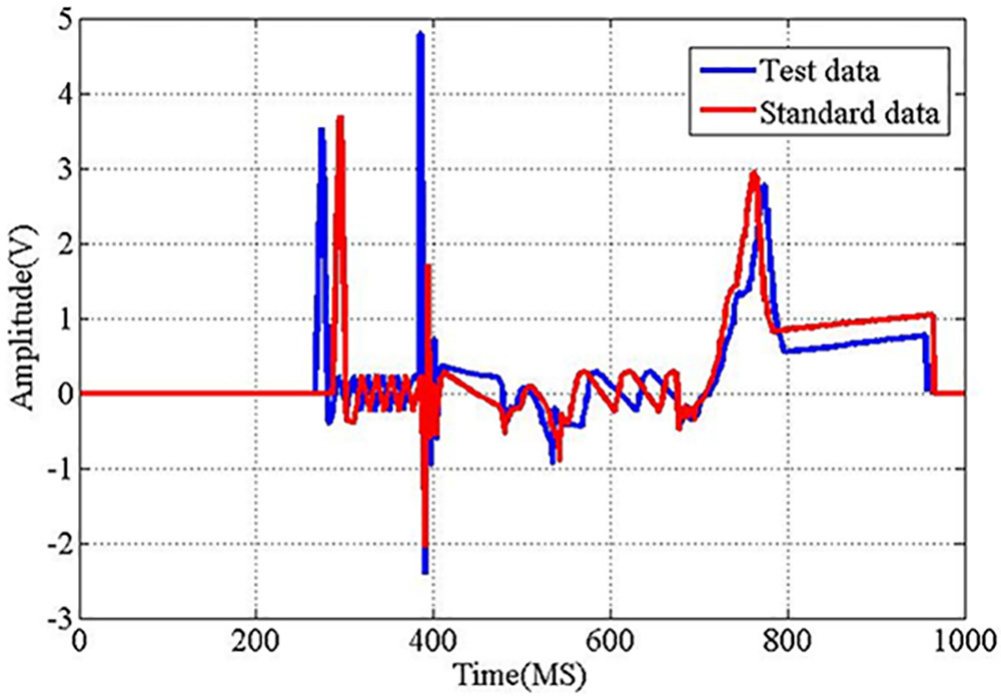
Test data and standard data. The essence of the spacecraft electrical characteristics identification is to contrast the test signal with the standard signal. Comparing the test signal with the standard signal and obtaining the standard signal from multiple complex signals are two primary problems in the identification of spacecraft electrical characteristics. The typical test data sample and a typical standard data sample obtained from the electrical characteristics of the spacecraft are shown in the Fig.

Standardization of similarity can be converted to construct an error function between test data and standard data. Both typical test sample and standard sample are shown to identify the standard similarity (Fig 1).

The error function can be used to hunt for the greatest similarity, which is given as follows:

$$\min_{W,T}\beta ={∥WD_{t}+T-D_{s}∥}_{2}\;$$ (1)

where the test data *D*_*t*_ and the standard data *D*_*s*_ represent the input of the Spacecraft electrical characteristics, *W* is a mapping transformation, and *T* is translation. The mininum *β* means the maximum similarity. The test data *D*_*t*_ and standard data *D*_*s*_ are provided in S1 Dataset.

### B.The off-line system and fuzzy clustering methods

The identification system of spacecraft electrical characteristics is including: the off-line system and the on-line system. Once the standard of similarity is identified, standard signals can be obtained from a large and complex signal dataset, which is very complex and time-consuming. an off-line fuzzy c-means clustering algorithm has been proposed for resolving these problems, which have been discussed in our previous works [31, 32].

In the off-line identification system, the electrical characteristic data are as inputs to the first pre-processing operation, which includes the down-sampling, filtering and event trigger threshold. After the generation of pretreatment isometric data with FCM clustering, the off-line system obtains a preliminary sample marked a labeled expert training dataset, which is an online identification system with entering parameter.

**Fig 2:**
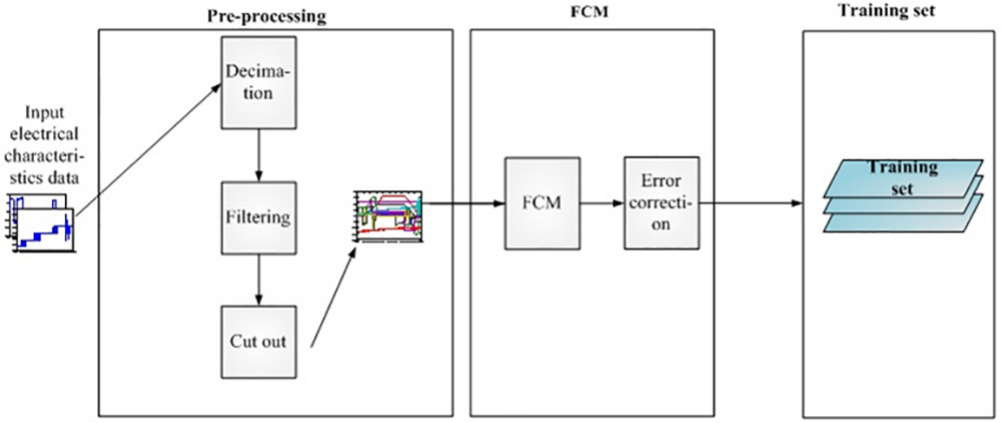
Flow chart of the off-line system. The off-line system gets a large number of signal data as input. These signal data are first processed by the pre-process procedure. The pre-process aims to divide a time series into several events by threshold value. When the time series traversal is over, the off-line system will put all of these samples into the fuzzy c-means section. The fuzzy c-means clustering is a process to produce the training set. This process clusters similar waveform data together and labels those waveforms to find the same category, then generates a clustering center for every class.

In this section, the off-line system gets a large number of input signals. The historical original signals are a long time series. Before the clustering algorithm is used to label the historical original signals, a pre-process has been used for the original data, where the pre-process aims to divide a time series into several events by threshold. If the signal is over threshold, an event has been triggered, and then a sample will be cut out from the time series by using a fixed length window. If the time series traversal is over, the off-line system will put all of these samples into the fuzzy c-means section. The historical original signal data are the inputs to the off-line system, and the training set is the outputs of the off-line system. The off-line system flow chart is given in Fig 2.

The fuzzy c-means clustering is applied to produce the training dataset. This process is including the clusters with similar waveform data together and labels those waveforms to find the same category, then a clustering center can be generated for each class. Finally, expert data inputs for error correction can be obtained.

**Fig 3:**
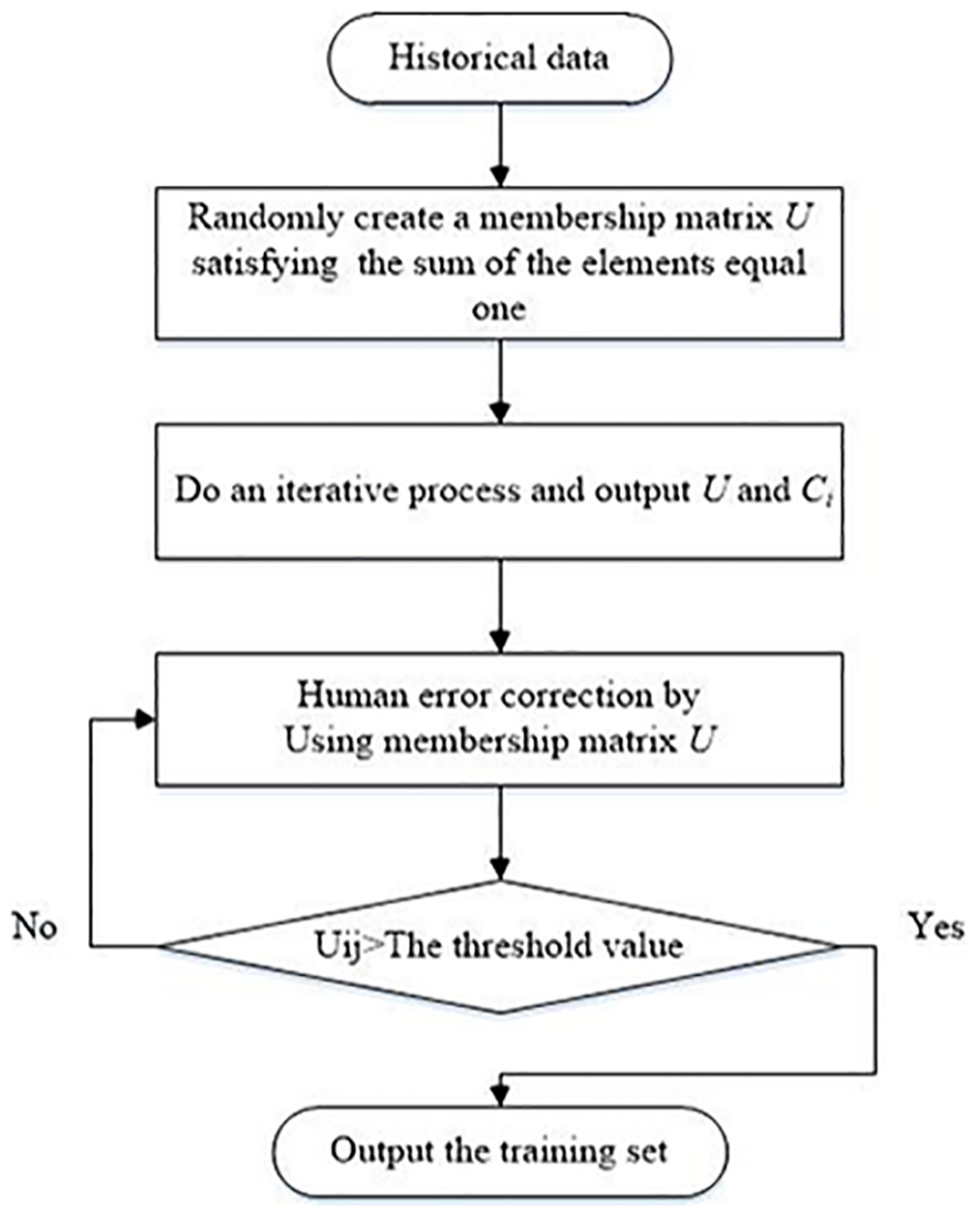
Flow chart of the FCM used in our article. The steps of fuzzy c-means clustering have several sections: first, the aim of fuzzy c-means clustering is to divide n samples into a fuzzy set. Second, each group’s clustering center can be calculated by minimizing the value function. Third, fuzzy c-means gives each sample a membership value between 0 and 1 to indicate its degree of membership to each cluster. It is a typical iterative process. Through the clustering algorithm, the off-line system generates the spacecraft electronic load historical data clustering, and gets the clustering center for events.

The fuzzy c-means clustering algorithm is an iterative process [33–37]. The flow chart of FCM in this paper is shown in Fig 3. The steps of fuzzy c-means clustering are including: 1), the aim of fuzzy c-means clustering is to divide n samples into a fuzzy dataset; 2), each group clustering center can be calculated by minimizing the error function; 3), fuzzy c-means gives each sample a membership value between 0 and 1, which indicates its degree of membership to each cluster.

In the process of clustering, the fuzzy c-means clustering function is:

$$minJ\left(U,c_{1},...,c_{c}\right)=\sum_{i=1}^{c}J_{i}=\sum_{i=1}^{c}\sum_{j=1}^{n}\mu_{ij}^{m}d_{ij}^{2}$$ (2)

$$\begin{array}{cc} min\bar{J}(U,c_{1},...,c_{c},\lambda_{1},...,\lambda_{n}) & =J(U,c_{1},...,c_{c})+\sum_{j=1}^{n}\lambda_{j}(\sum_{i=1}^{c}\mu_{ij}-1)\\ & =\sum_{i=1}^{c}\sum_{j}^{n}\mu_{ij}^{m}d_{ij}^{2}+\sum_{j=1}^{n}\lambda_{j}(\sum_{i=1}^{c}\mu_{ij}-1)\; \end{array}$$ (3)

where Eq (2) is the transformation of Eq (3), the *λ*_*ij*_ is Lagrange multiplier, minimizing the function can be calculated as follows:

$$\begin{array}{c} c_{i}=\frac{\sum_{j=1}^{n}\;\mu_{ij}^{m}x_{j}}{\sum_{j=1}^{n}\;\mu_{ij}^{m}},\mu_{ij}=\frac{1}{\sum_{k=1}^{c}{\left(\frac{d_{ij}}{d_{kj}}\right)}^{2/(m-1)}}\\ s.t.\sum_{i=1}^{c}\mu_{ij}=1,\forall j=1,...,n \end{array}$$ (4)

where *c*_*i*_ is the clustering center of fuzzy set *i*, *μ*_*ij*_ is a member of membership matrix *U*, *d*_*ij*_ = ||*c*_*i*_ − *y*_*j*_|| is the Euclidean distance between the *c*_*i*_ clustering center and the *c*_*i*_ and *y*_*j*_ sample data, *x*_*j*_ is an electrical characteristics input sample, and *m* is a weighted index.

Through the clustering algorithm, the off-line system generates the spacecraft electronic load historical data clustering, and gets the clustering center for events. Compared with general clustering methods, the fuzzy c-means clustering method obtains a membership matrix, which means that no definite samples of historical data need to be error- corrected, which greatly reduces the manual work.

## III. On-line identifiction system

### A.Summary

Once obtaining an expert training dataset from the off-line system, the training dataset is regarded as one input to the on-line identification system. Another input to the on-line system is a real time on-line data, which needs to be identified as well.

**Fig 4:**
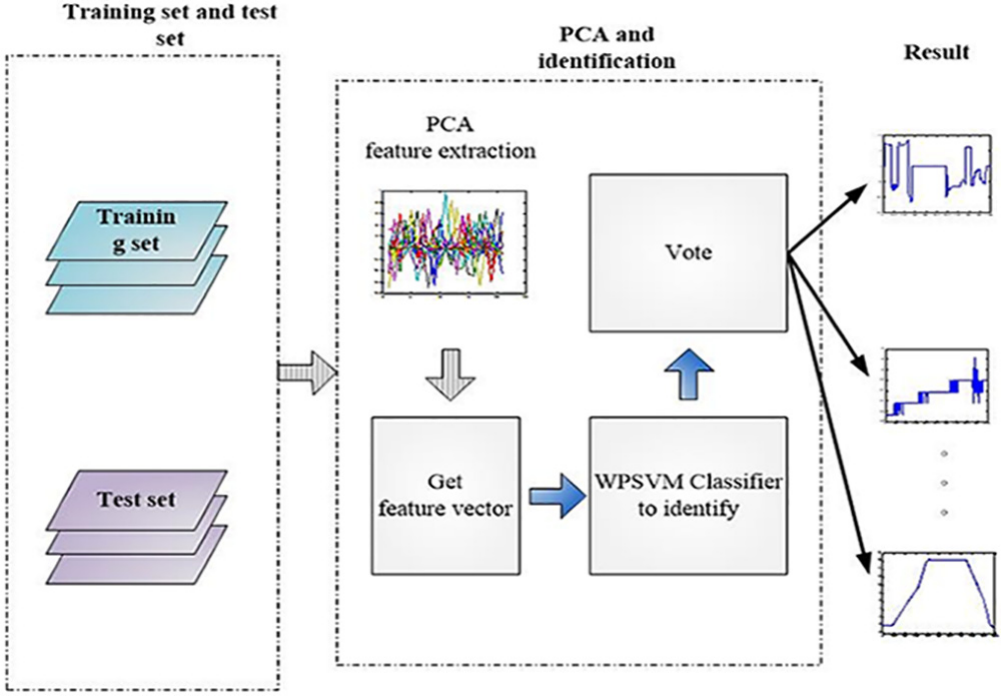
On-line electrical characteristics identification general flow-chart. The method using PCA feature extraction and the WPSVM classifier to providing electrical characteristics identification in the spacecraft load system is proposed. The on-line identification process uses the first training set generated off-line and the online-generated test as input. The PCA dimensionality of these data are reduced. After obtaining the PCA feature extraction vector, the input feature vector classifier is used to provide classification. Vote treatment in the presence of multiple classifiers is used to obtain the final classification result.

The PCA feature extraction and the WPSVM classifier method are proposed to provide electrical characteristics identification in the spacecraft load system. An on-line electrical characteristics identification is shown in Fig 4. The on-line identification process is used to the first training dataset by generated offline and the online-generated test as input. The PCA dimensionality of these data are reduced. After obtaining the PCA feature extraction vector, the input feature vector classifier is used to provide classification. Vote treatment in the presence of multiple classifiers is used to obtain the final classification result.

This flow-chart is composed of several processes. The PCA feature extraction is firstly used in the category model to reduce the feature vector, which could be used by WPSVM classifiers. The n WPSVM classifiers are then used to obtain n results to identify the electrical characteristics. Finally, a rank is used to summarize the n results into one, and thus the on-line signal data are identified. The details of the method will give in the next section.

### B.PCA feature extraction method

PCA is also called feature extraction method of the primary component analysis, which is a data dimension reduction process based by calculating the statistical covariance matrix of the spacecraft’s electronic load electrical properties [38–40]. Its purpose is to find those elements which contribute most to the features of the data. The little changed elements can be got rid of, thus reducing the dimensionality so that the amount of calculation can be reduced [38, 39, 41–43].

**Fig 5:**
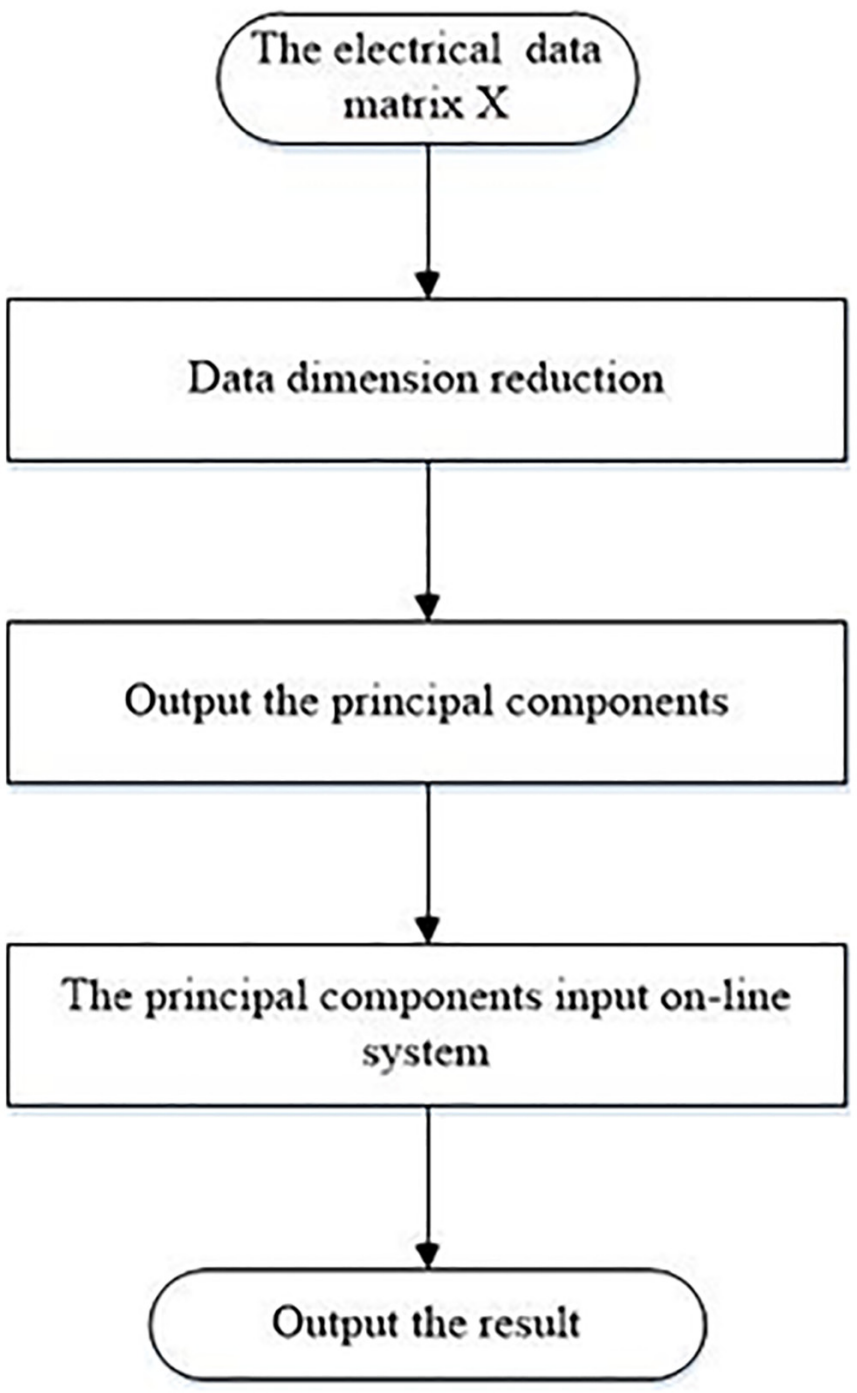
Flow chart of PCA in article. The steps of PCA feature extraction method have several sections: first, there are electrical data matrix X as input. Second, through the data dimension reduction, then output the principal components. Third, entering the principal components into online system, then output the result. PCA is a data dimension reduction process based on calculating the statistical covariance matrix of the spacecraft’s electronic load electrical properties. Its purpose is to find those elements which contribute most to the features of the data. The little changed elements can be got rid of, thus reducing the dimensionality so that the amount of calculation can be reduced.

The flow chart of the PCA feature extraction method used in our article is detailed in Fig 5.

Define each section of the spacecraft electrical characteristics data as vectors in the form of:

$$X=[X_{1},X_{2},\ldots \ldots ,X_{n}]$$ (5)

*X*_*k*_ = (*x*_*k*,1_, *x*_*k*,2_, ⋯, *x*_*k*,*r*_) (1 ≤ *k* ≤ *n*) stands for the k samples, and r is the number of point of the sample, n is the number of sample. Then, calculate the covariance matrix S is as follows:

$$\begin{array}{c} S=\frac{1}{n}\sum_{k=1}^{n}\left(X_{k}-\bar{X}\right){\left(X_{k}-\bar{X}\right)}^{T}\\ s.t.\bar{X}=\frac{1}{n}\sum_{k=1}^{n}X_{k} \end{array}$$ (6)

$\bar{X}$ is the avenge value of samples. *S* is a r*r matrix, by calculating the eigenvalues of S which are [*λ*1, *λ*2, ……, *λn*](*λ*_1_ ≥ *λ*_2_ ≥ ……≥*λ*_*n*_), then the eigenvector *T* = [*u*_1_, *u*_2_, ……, *u*_*n*_] should be obtained, the eigenvector is the orthogonal basis for the spacecraft electrical data [43]. Greater feature value can make greater contribution. Calculating the contribution using normalized methods, the contribution *P*_*k*_ could be measured as follows:

$$Pk=\lambda k{\left.(\sum_{i=1}^{n}\lambda i\right)}^{-1}$$ (7)

Those eigenvectors that have tiny feature value should be ignored. Using the first d vectors of the matrix X to restore the model, where $\sum_{i=1}^{d}P_{i}^{}\geq P$, the restore matrix $\hat{X}$ is as follows:

$$\hat{X}=\sum_{i=1}^{d}u_{i}^{T}Xu_{i}$$ (8)

By calculating the covariance matrix to identify the elements that have larger covariance, these elements have much more weight on the spacecraft electrical property data than elements which have smaller covariance. This provides a way to realize dimensionality reduction, thus greatly accelerating the computation speed.

### C.Weighed proximal support vector machine

In our experiments, we use the following method to train and to verify the accuracy of the classifier. This method divides the data set into two different sets referred as the training set and the test set. Then, the classification model is trained using the training set, which means we can use the test set for validation. In the process of our experiments, the training set is the feature extraction of expert data, and the test set is the feature extraction of online data.

The SVM classifier is the classifier we used in the identification process, which is based on statistical learning VC dimension theory and the structure risk minimum principle [44, 45]. In the process of recognition, by mapping the spacecraft electrical properties data to achieve linear classification, the aim of the SVM model is to maximize the interval. The weighed proximal support vector machine algorithm is as follows:

$$\begin{array}{c} \underset{}{max}\delta m=\frac{1}{\left|\omega \right|}\\ s.t.y_{i}\left(\omega •{\hat{x}}_{i}+b\right)-1\geq 0\left(i=1,2,\ldots \ldots l\right) \end{array}$$ (9)

*δm* is the hyperplane interval, ${\hat{x}}_{i}$ is the sample characteristics obtained, *ω* and *b* are respectively, the normal vector and displacement of the hyperplane.

Using WPSVM to identify the spacecraft electrical characteristics, and comparing SVM with WPSVM, it changes the problem to another function as follows:

$$\begin{array}{c} \underset{}{m\text{in}}\left(\frac{1}{2}{\left|\omega \right|}^{2}+\frac{1}{2}\sum_{i=1}^{d}C_{i}s_{i}\xi_{i}^{2}\right)\\ s.t.y_{i}\left(\omega •{\hat{x}}_{i}+b\right)-1+\xi_{i}=0\left(i=1,2,\ldots \ldots l\right) \end{array}$$ (10)

*C*_*i*_ represents a parameter that is different to positive and negative classes; *ξ*_*i*_ is the slack variable; *d* is the sample size; *ω* is the normal vector of the optimal hyperplane; *b* is the displacement; ${\hat{x}}_{i}$ is a sample vector; *y*_*i*_ is the ${\hat{x}}_{i}$ category id. *s*_*i*_ is the value of an adjustable parameter for each training sample, standing for the contribution of each type, 0 ≤ *s*_*i*_ ≤ 1.

To solve the above optimization problem, the Lagrange theorem is introduced. The problem can be converted as follows:

$$\begin{array}{c} \underset{\omega ,b,\xi ,\alpha }{m\text{in}}L\left(\omega ,b,\xi ,\alpha \right)=\frac{1}{2}{\left|\omega \right|}^{2}+\frac{1}{2}\sum_{i=1}^{d}C_{i}s_{i}\xi_{i}^{2}\\ -\sum_{i=1}^{d}\alpha_{i}\;\left[y_{i}\left(\omega •{\hat{x}}_{i}+b\right)-1+\xi_{i}\right] \end{array}$$ (11)

*α* = (*α*_1_, *α*_2_, …… *α*_d_) is the Lagrange multiplier. By using Wolfe Duality theorem [41, 42] to calculate the minimum *L*(*ω*, *b*, *ξ*, *α*) define by *ω*, *b*, *ξ*, *α* as follows:

$$\frac{\partial L}{\partial \omega }=\omega -\sum_{i=1}^{d}\alpha_{i}y_{i}{\hat{x}}_{i}=0$$ (12)

$$\frac{\partial L}{\partial b}=\sum_{i=1}^{d}\alpha_{i}y_{i}=0$$ (13)

$$\frac{\partial L}{\partial \xi }=\sum_{i=1}^{d}C_{i}s_{i}\xi_{i}^{2}-\sum_{i=1}^{d}\alpha_{i}=0$$ (14)

$$\frac{\partial L}{\partial \alpha }=y_{i}\left(\omega •{\hat{x}}_{i}+b\right)-1+\xi_{i}=0$$ (15)

Using *δ*_*i*_ = *C*_*i*_ *s*_*i*_ to Eqs (12)–(15) and solving the problem, it follows that:

$$\left\{\begin{array}{c} \omega =\sum_{i=1}^{d}\alpha_{i}y_{i}{\hat{x}}_{i}\\ \sum_{i=1}^{d}\alpha_{i}y_{i}=0\\ \sum_{i=1}^{d}\delta_{i}\xi_{i}^{2}=\sum_{i=1}^{d}\alpha_{i}\\ y_{i}\left(\omega •{\hat{x}}_{i}+b\right)-1+\xi_{i}=0 \end{array}\right.$$ (16)

Making Eq (13) into Eq (8) to solve the dual problem, and obtaining the optimal solution $\alpha *=({\alpha_{1}}^{*},{\alpha_{2}}^{*},\ldots \ldots \alpha_{d}^{*})$, choosing a positive α* where α* > 0, then the solution of original problem can be represented as follows:

$$\omega^{*}=\sum_{i=1}^{d}{\alpha^{*}}_{i}y_{i}{\hat{x}}_{i}$$ (17)

$$b^{*}=y_{j}\left(1-\frac{\alpha_{i}}{\delta_{i}}\right)-\sum_{i=1}^{d}{\alpha^{*}}_{i}y_{i}K\;\left({\hat{x}}_{i},\hat{x}j\right)=0$$ (18)

$K({\hat{x}}_{i},\hat{x}j)$ means kernel function, which is used for mapping the input space to the corresponding high dimensional dimensional feature space. Using different kernel functions may make a small difference. The final decision function is:

$$F\left(\hat{x}\right)=sgn\left(\omega^{*}•\hat{x}+b^{*}\right)$$ (19)

In this paper, we employ the radial basis kernel function as the Gauss kernel function [46, 47], which is given as follows:

$$K\left(x,xf\right)=exp\left(\frac{\left||x-xf\right|{|}^{2}}{{\left(2\delta \right)}^{2}}\right)$$ (20)

Based on the WPSVM, we can identify a classifier model for spacecraft electrical characteristics identification.

### D.Similarity calculation formula

$$\left\{\frac{\sum \left(\left|D_{t}-D_{s}\right|•sgnT\right)}{\text{Size}D_{t}}\right\}$$ (21)

Similarity using the formula is shown in the prompt box. *D*_*t*_ representative test samples, and *D*_*s*_ represents the standard sample. To sign function that set the threshold T is sgn(T). The sample Size (*D*_*s*_) represents the number of sample data points, that is, the dimension.

## IV.Experiment Results

**Fig 6:**
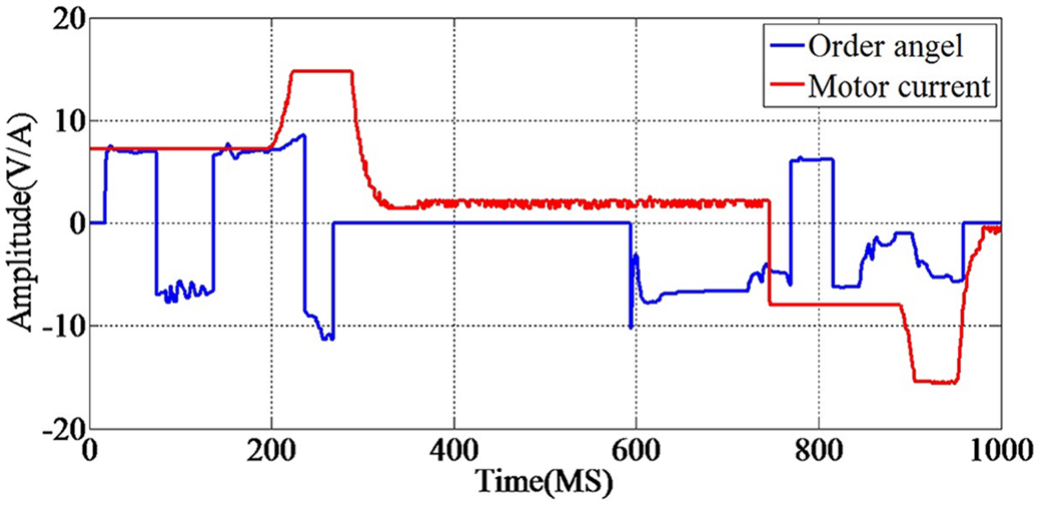
Typical electrical characteristics data. Simulation experimental data comes from the spacecraft electronic load for a typical experiment, the original data for each section are 1000 data points’ waveforms.

**Fig 7:**
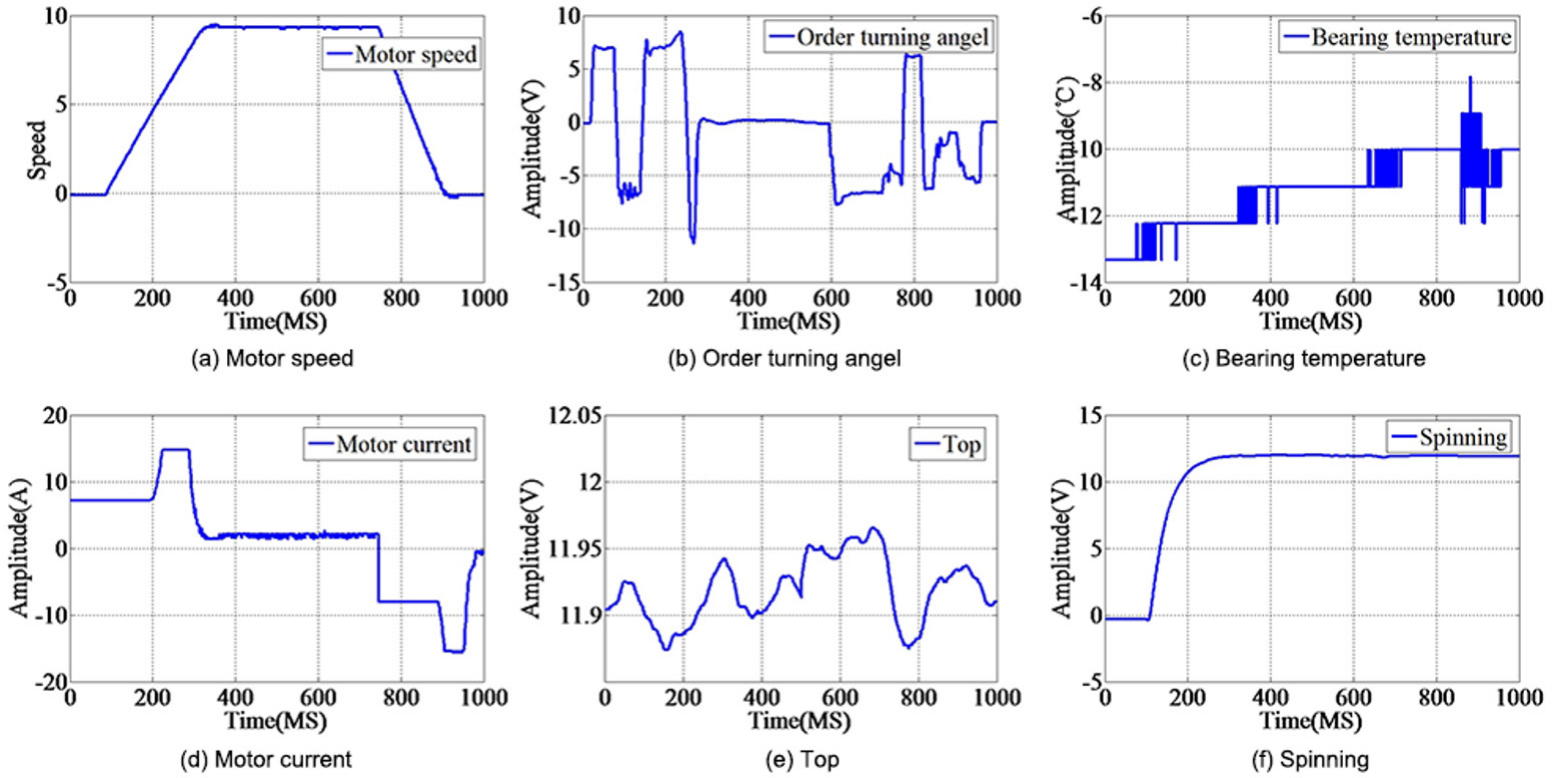
Random electrical characteristics data sample. There are 10, 50, 100, and 500 samples randomly extracted from the real data set, combining six elements of the physical process: (a)motor speed, (b)order turning angle, (c)bearing temperature, (d)motor current, (e)top and (f)spinning.

Simulation data comes from the spacecraft electronic load for a typical experiment, the original data for each section are 1000 data points waveforms (Fig 6). Typical data combine motor speed, order turning angle, bearing temperature, motor current, top and spinning (Fig 7). The simulation data and typical dataset are provided in S1 Dataset

The experiments can be briefly summarized as follows. First, the FCM clustering algorithm is used to preliminarily label samples, whereby standard data can be obtained as the training set. Second, the feature extraction method based on PCA is used to reduce the big data dimension. Finally, the WPSVM classifier is used to obtain the identification test set and improve calculation accuracy.

The experiments are conducted using Matlab on an Intel (R) Core (TM) i7-3520M CPU @ 2.90 GHz, 8.0 GB.

### A.Comparision of the training speed using PCA and without PCA

Before the on-line algorithm test, the FCM algorithm was used to obtain the clustering centers and to obtain the membership matrix U at the same time.

**Fig 8:**
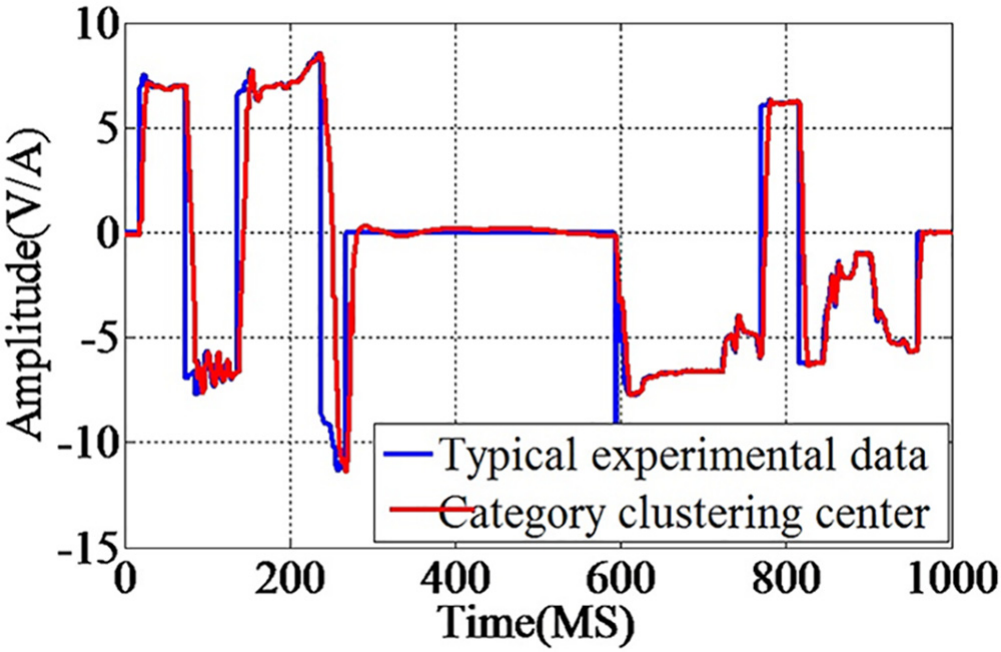
Spacecraft electronic load typical experimental data and the category clustering center. Before the on-line algorithm test, the FCM algorithm was used to obtain the clustering centers. An example of a roll event during the spacecraft load test experiment is detailed in the Fig.

**Fig 9:**
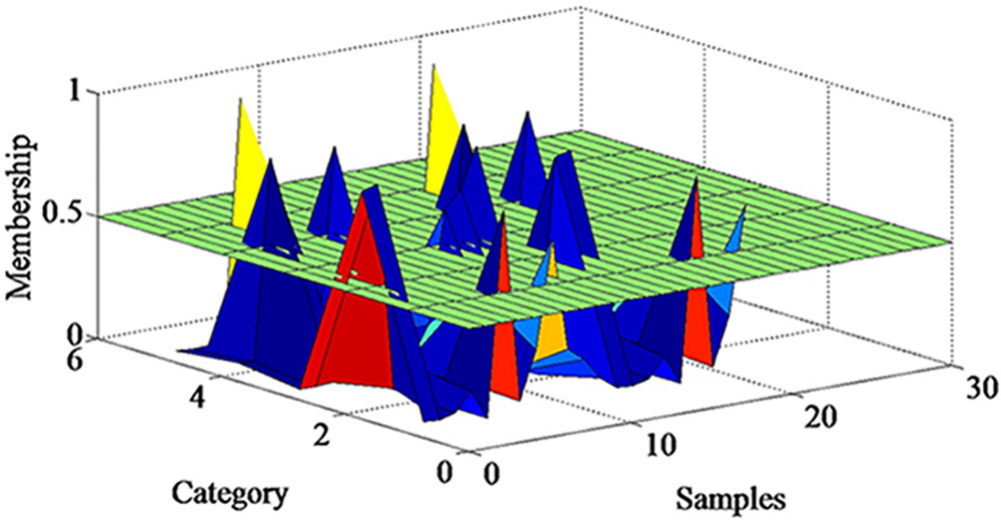
Membership matrix U. The sample that has high membership can be confirmed directly; other samples which belong to multiple categories with similar membership require further revision by human experts. Compared with k-means algorithm, the FCM algorithm offered an extra matrix U which uses a threshold set by us. Only the elements of the matrix U which are less than the threshold need to be corrected by humans. The matrix U will save much human error correction time in the off-line system.

An example of a roll event during the spacecraft load test experiment is detailed in Fig 8. The membership matrix U is shown in Fig 9. Compared with k-means algorithm, the FCM algorithm offered an extra matrix U which uses a threshold set by us. Only the elements of the matrix U which are less than the threshold need to be corrected by humans. The matrix U will save much human error correction time in the off-line system.

The sample that has high membership can be confirmed directly; other samples which belong to multiple categories with similar membership require further revision by human experts. After FCM and human error correction, the basic training data are ready for use. These data are referred to as expert training data. The category model proposed by this article has two inputs, training data and test data, aiming to identify the test data. In this category model, we use PCA feature extraction to make a dimension reduction at first. In this experiment, the first 21 component represented over 90% of the weights. Therefore, using 21 components as features is adequate and feasible.

The testing of the training time with PCA and without PCA by using different number of samples such as 10, 50, 100 and 500 is detailed in (Table 1). The results shows that with the PCA feature extraction the calculation time is obviously reduced (Table 1). In this way, the calculation speed can satisfied within the design requirements and can enable on-line identification. The identification results of several methods will be shown in the following paragraph.

**Table 1:** The calculate time comparison.

**Feature extraction methods**	**10 samples/s**	**50 samples/s**	**100 samples/s**	**500 samples/s**
				
*PCA*	0.12292	0.313488	0.532109	1.020358
*WithoutPCA*	1.73184	3.833223	7.583167	19.364252

### B.The accuracy and speed by using WPSVM in this article

After completion of clustering and PCA feature extraction, the clustering center has been found and can be used in identification. Use of the WPSVM classifier is the next step in the category model. The WPSVM classifier has a two category classifier; to make a multi-class classifier, we must use several WPSVM classifiers. There are two solutions to this multi-class problem. Those two solutions are named one-to-one methods and one-to-the-other methods. One-to-one methods means that every pair of categories has a WPSVM classifier; for n categories, there are *n*(*n* − 1)/2 classifiers. In this model, each classifier will vote on one category; the category which obtain the most votes is the final category.

One-to-the-other methods means one category and the other category each have a WPSVM classifier; for n categories there are n classifiers. In this model, if there is only one category that received votes, this category is the final category. If more than one category received votes, comparing the outputs $\omega^{*}•\hat{x}+b^{*}$, the biggest output is the result.

**Fig 10:**
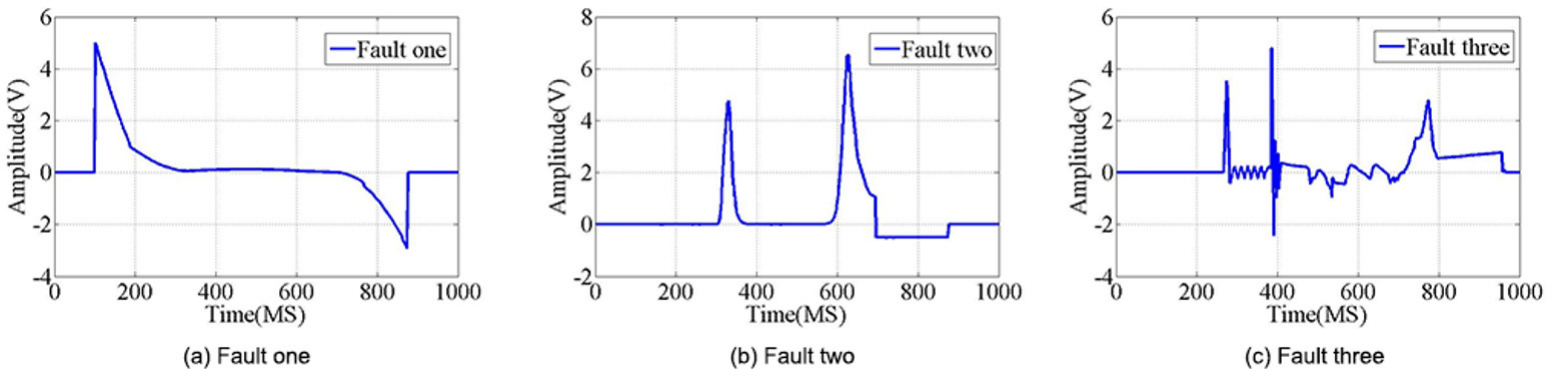
Random electrical characteristics data sample. There are 10, 50, 100, and 500 samples randomly extracted from the real data set, combining three categories of typical fault signals, (a)Fault one, (b) Fault two, (c)Fault three.

**Fig 11:**
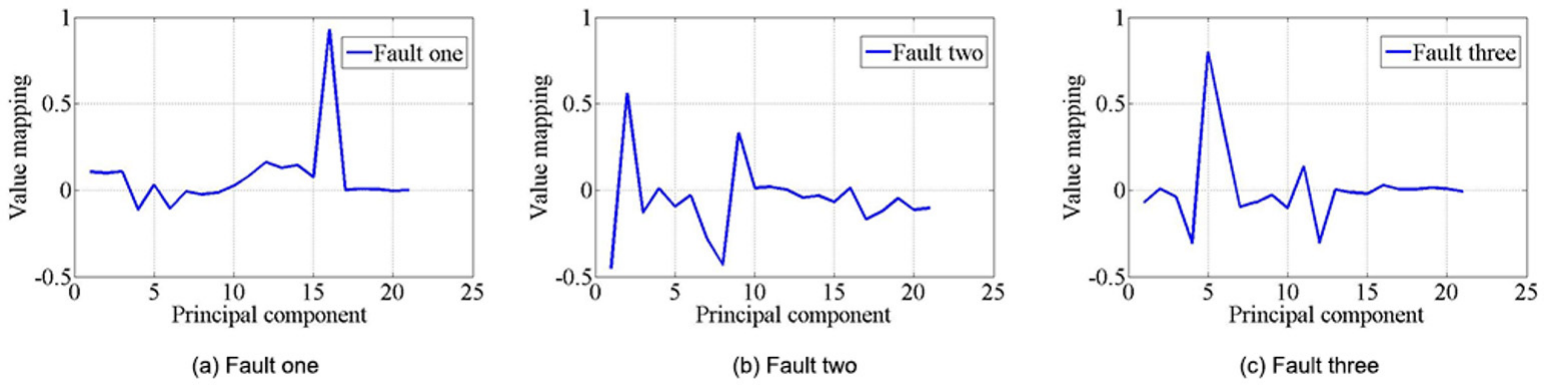
Random electrical characteristics data sample dimension reduction by PCA. The typical fault signals of randomly chosen real data sample and the samples’ dimension reduction by PCA are detailed in the Fig. (a)Fault one, (b) Fault two, (c)Fault three.

**Fig 12:**
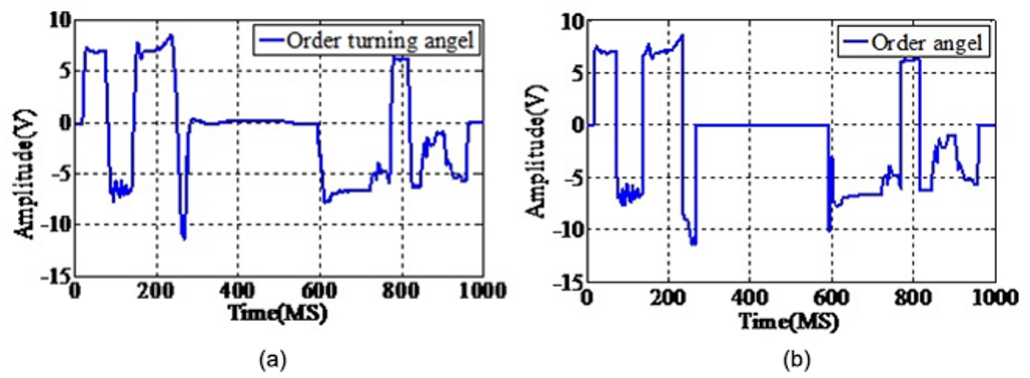
A typical example of calculating the similarity. The standard of similarity is identified. We recorded the training time and the accuracy of each method. We changed the parameters of each method, and the highest accuracy was recorded as the result for each method. which the threshold is set as 0.1 and the similarity result is 95%.

The experiment in this paper used 10, 50, 100, and 500 samples randomly extracted from the real data set, combining six elements of the physical process: motor speed, order turning angle, bearing temperature, motor current, top and spinning (Fig 7), and three categories of typical fault signals are shown in Fig 10. We recorded the training time and the accuracy of each method. We changed the parameters of each method, and the highest accuracy was recorded as the result for each method. Finally, the results of each method are compared with each other. The typical fault signals of randomly chosen real data sample and the samples’s dimension reduction by PCA are detailed in Figs 11 and 12, on which the threshold is set as 0.1 and the similarity result is 95%. These data are the input to the next simulation experiments.

We can see that, for multi-label classification, using the SVM algorithm has higher accuracy than the Bayesian algorithm and the KNN algorithm (Table 2). And compared with the SVM algorithm, the WPSVM algorithm has obvious accuracy improvement. The one-to-one WPSVM algorithm has better results is than the one to other WPSVM algorithm. However, each algorithm has difficulty solving the problem of identification, with greater difficulty for the samples with higher tensile.

**Table 2:** The comparison of calculate time.

**Feature extraction methods**	**10 samples**	**50 samples**	**100 samples**	**500 samples**
Bayes	0.6	0.64	0.66	0.65
KNN	0.7	0.70	0.72	0.71
ONE TO ONE SVM	0.9	0.88	0.9	0.9
ONE TO ONE WPSVM	0.9	0.92	0.93	0.93
ONE TO OTHERS SVM	0.9	0.88	0.89	0.89
ONE TO OTHER WPSVM	0.9	0.90	0.92	0.91

**Fig 13:**
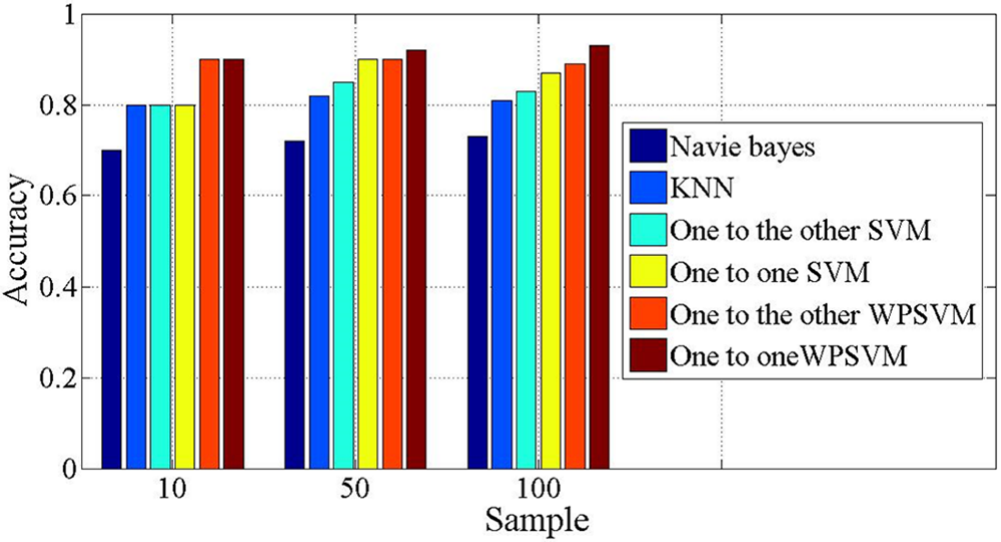
Classification accuracy use different algorithm. Naive-Bayes algorithm, the KNN algorithm, one-to-one SVM, one-to-the-other SVM, one-to-one WPSVM and one-to-the-other WPSVM are used in the on-line system. It shows the accuracy of test data identification.

The feature extraction simulation experiment is conducted similar to the PCA simulation experiments. The identification of accuracy for test data in the on-line system using different methods are given (Fig 13), which include the naive-Bayes algorithm, the KNN algorithm, one-to-one SVM, one-to-the-other SVM, one-to-one WPSVM and one-to-the-other WPSVM. The conclusion is that the WPSVM algorithm that we propose has better accuracy than the classical algorithms. When the sample number is 100, the classification performance is more obvious.

We further observe the registration situation of SVM and WPSM for identification accuracy (Fig 13). It can be seen that SVM and WPSVM have the same accuracy in the first stage of 10 samples, but when the sample sets are large enough, the SVM algorithm will give a lower accuracy than WPSVM. In the WPSVM method, the more effective points can obtain greater weights. In contrast, the points which are noise can obtain smaller weight. In this way, it effectively reduces the impact of noise on the identification results and makes the final identification result more accurate. The result of the WPSVM algorithm compared with the classical naive Bayes algorithm, the SVM algorithm, and the KNN algorithm are detailed in Fig 13 and Table 2. It also can be seen that, using the WPSVM, the recognition accuracy has obviously improved.

## V.Conclusion

This article used unsupervised clustering that is not dependent on the predefined categories of classes, proposed an improved fuzzy c-means clustering algorithm to label initial data and then obtained the clustering center by error correction. After using PCA feature extraction and the weighted proximal support vector machine (WSVM) algorithm proposed in this article to resolve the problem of electrical characteristics identification, eventually we improved the precision of identification, and further enhanced the performance of the classifier.

For this model, the method proposed solves the problems of large amounts of unlabeled test data, high dimension characteristics, slow computing speed and low recognition rate during the process. Our further work is to solve the sensitive problem of abnormal overlay events, which are also difficult to identify. Other problems are minimizing the influence of dispersion data or isolated points, reducing artificial intervention and not adding too much computational complexity at the same time. We propose to test the effectiveness of our proposed method with different large scale data sets, in order to construct a classifier with better performance in a spacecraft electrical characteristics identification system. The results of this research can be used in spacecraft fault diagnosis systems in the future.

## Supporting Information

S1 Dataset Including the Standard Data, Test Data, Simulation Data and Typical Data. (RAR)
